# Phosphorescent Sensor Based on Iridium(III) Complex with Aggregation-Induced Emission Activity for Facile Detection of Volatile Acids

**DOI:** 10.3390/molecules29246041

**Published:** 2024-12-22

**Authors:** Yu Pei, Yan Sun, Dongxia Zhu

**Affiliations:** Key Laboratory of Nanobiosensing and Nanobioanalysis at Universities of Jilin Province, Department of Chemistry, Northeast Normal University, 5268 Renmin Street, Changchun 130024, China; peiy121@nenu.edu.cn (Y.P.); suny902@nenu.edu.cn (Y.S.)

**Keywords:** Iridium(III) complex, aggregation-induced emission (AIE), acid-base stimulation

## Abstract

Phosphorescent sensors are essential for rapid visual sensing of volatile acids, due to their profound impact on ecosystems and human health. However, solid phosphorescent materials for acid-base stimulus response are still rare, and it is important to achieve real-time monitoring of volatile acids. In order to obtain an efficient and rapid response to volatile acid stimulation, N-H and -NH_2_ substituents are introduced into an auxiliary ligand to synthesize a new cationic Ir(III) complex (**Ir-NH**). The AIE property of **Ir-NH** leads to enhanced emission in the aggregated state, which facilitates the construction of solid-state acid-base sensors. More importantly, due to the introduction of -NH_2_ and N-H in the molecular structure, reversible switching of the emission color of **Ir-NH** under acid-base stimulation was successfully achieved. A convenient and efficient sensing device for volatile acid monitoring was prepared using **Ir-NH** as the active material. Our results provide a new strategy for designing phosphorescent materials with AIE and acid-base stimulus-responsive properties.

## 1. Introduction

Acids and bases, as crucial, basic chemical raw materials, are widely used in agricultural and industrial production, and the release of their corresponding gasses is on the rise. As corrosive substances, volatile acids are severe ecological pollutants. They cause environmental changes such as acid rain and also affect human health; for example, by causing chemical burns, bronchitis and pulmonary edema [1,2,3]. Real-time monitoring of volatile acids is essential in both production and life. Volatile acids are generally detected using standard analytical methods such as gas chromatography, electrochemical analysis, and UV-visible absorption spectroscopy [4,5,6,7,8]. However, these methods usually have the disadvantages of expensive instrumentation, inconvenient portability, and complicated operation, which are not conducive to rapid real-time monitoring of volatile acid generation. In recent years, chemical sensors have received much attention. They converts chemical stimuli into signals that can be easily observed by the naked eye, such as electronic signals or switching color changes in fluorescence [9,10]. Therefore, using simple and low-cost chemical sensors for detecting acid gasses is of great importance. Wu et al. reported a sensor made of porphyrin polyimide nanofiber membrane for detecting acidic gasses [11]. Zhao’s group reported a COF material with simultaneous sensing of acidic and alkaline vapors with obvious colorimetric sensing [12]. Among these reported sensors, paper-based sensors are the most convenient and portable [13,14]. Attaching acid-base responsive solid dyes to paper can effectively observe the change in fluorescence brightness or color. However, there are few reports on paper-based sensors, mainly due to the aggregation-caused quenching (ACQ) phenomenon, which leads to a decrease in the sensitivity of the sensors [15]. It seriously limits the practical application of solid-state luminescent materials in the detection field. 

Aggregation-induced emission (AIE) refers to molecules that emit weak or even no emission in the solution state and enhanced emission in the aggregated or solid state [16,17]. The introduction of this concept has dramatically facilitated the application of solid-state luminescent materials in the field of chemical sensors [18,19]. Many AIE-based fluorescent sensors have been reported; Tian et al. constructed a classical tetraphenylethene (TPE)-functionalized spiropyran (SP) molecule with AIE and acid-base stimulus-responsive properties [20] and Liu and his colleagues developed a series of NBI-based acid-chromogenic AIEgens and prepared acidity monitoring chips [21]. Compared to the rapidly developing fluorescent AIE sensors, there are relatively few reports of phosphorescent AIE sensors based on transition metal complexes [22,23,24,25,26]. Ir(III) complexes are potential molecules for constructing phosphorescent AIE sensors due to their large Stokes shift, good photostability, long phosphorescence lifetime, and distorted structure [27,28,29]. Therefore, it is a challenge to design Ir(III) complexes based on AIE properties to achieve sensitive detection of volatile acids. 

In general, acid and alkali sensors are designed for the following two main purposes: (i) introducing substituents such as amino groups, phenolic hydroxyl groups, or pyridine groups into the molecule, which are prone to protonation under the action of acids or bases, causing changes in the luminescent properties of the molecule [30,31,32,33]; (ii) identifying the structural changes in molecular ring opening or isomerization under acid-base stimulation, which affect the luminescence intensity or color of the molecule [34,35]. Specifically, aromatic amines and N-heterocyclic derivatives (indole, imidazole, pyrrole, etc.) are ideal reaction sites for acid-base detection, because these substituents are extremely sensitive to acids and have high sensitivity, with speedy rates of protonation and deprotonation in acid-base equilibrium [36,37]. Hence, we envision introducing acid-base recognition signals with amino and N-heterocyclic substituents into the auxiliary ligand of Ir(III) complex to construct ideal AIE phosphorescent detection materials for volatile acids. 

**Scheme 1:**
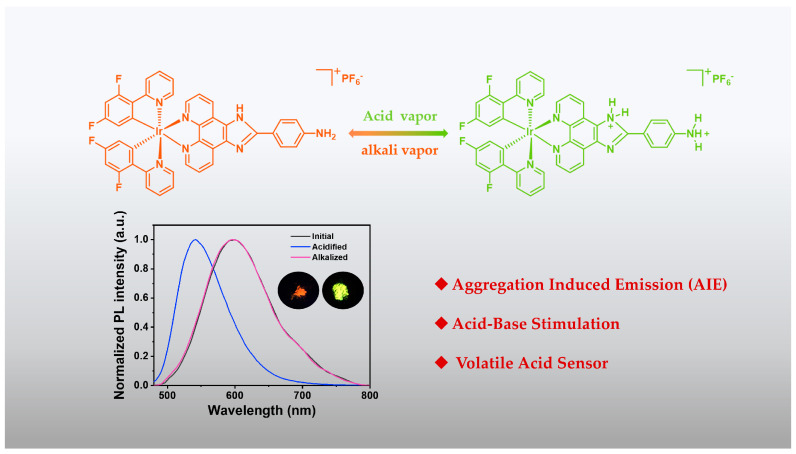
Acid-base response of **Ir-NH**.

Inspired by this idea, we designed and synthesized a novel cationic Ir(III) complex containing amino and N-heterocycles substituents, named **Ir-NH** (Scheme 1). The complex exhibited typical AIE property, and its solid powder showed a color change from orange (600 nm) to green (541 nm) under the stimulation of acidic and alkaline vapors, demonstrating excellent reversible cyclability. ^1^H NMR spectroscopy, FT-TR spectroscopy, and powder X-ray diffraction (PXRD) experiments revealed that the reversible acid-base response mechanism of **Ir-NH** could be attributed to the protonation and deprotonation processes of -NH_2_ and -NH substituents in the auxiliary ligand. Our molecular design strategy provides a clear direction for constructing acid-base stimulus-responsive phosphorescent AIE materials. Most importantly, by utilizing the reversible color change emitted by **Ir-NH** under acid-base stimulation, we have successfully prepared a convenient, fast, and recyclable paper sensor for monitoring volatile acids. This simple and visual sensing device is of great significance to ecosystems and human health.

## 2. Results and Discussion

### 2.1. Study of Photophysical Properties 

The photophysical properties of **Ir-NH** were investigated by UV-Vis absorption and emission spectroscopy in CH_3_CN solution, and the detailed data are presented in Table S1. As observed from the UV-Vis absorption spectra (Figure S5), the strong absorption band in the 300-350 nm interval was mainly attributed to the ligand-centered (^1^LC) π-π* transitions [38]. The moderately intense absorption bands in the 350 nm to the visible region can be ascribed to mixed metal-to-ligand charge transfer (MLCT) and ligand-to-ligand charge transfer (LLCT) transitions [39]. As shown in Figure S5, **Ir-NH** emits weakly in CH_3_CN solution under UV light, with a maximum emission wavelength of 602 nm.

### 2.2. Study of AIE Property 

**Figure 1:**
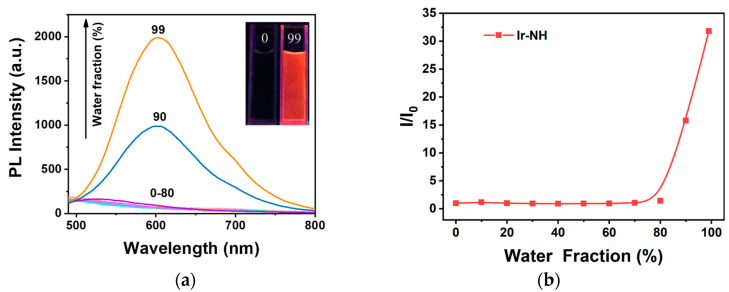
(**a**) The emission spectra of **Ir-NH** in water–CH_3_CN mixtures with different water fractions (0–99% v/v) at room temperature. Inset: emission image of **Ir-NH** in pure CH_3_CN solution and a water–CH_3_CN mixture (99% water fraction) under 365 nm UV illumination. (**b**) The plots of relative PL intensity (I/I_0_) of obtained **Ir-NH** versus different water fractions. I_0_ and I are the values of PL intensity at maximum peak in CH_3_CN and CH_3_CN/water mixtures, respectively.

Since the emission of **Ir-NH** was weak in solution and significantly enhanced in the solid state, we first explored its AIE properties. We added different fractions of water to the CH_3_CN solution of **Ir-NH** to study the emission changes. As shown in Figure 1, **Ir-NH** was very soluble in pure CH_3_CN solution, but the emission was weak. The emission intensity was significantly increased when the fraction of water reached 90%. When the water fraction reached 99%, the emission intensity of **Ir-NH** reached its maximum value, which was increased by more than 31 times. The above experimental phenomena indicate that **Ir-NH** is a typical phosphorescent material with AIE properties.

**Figure 2:**
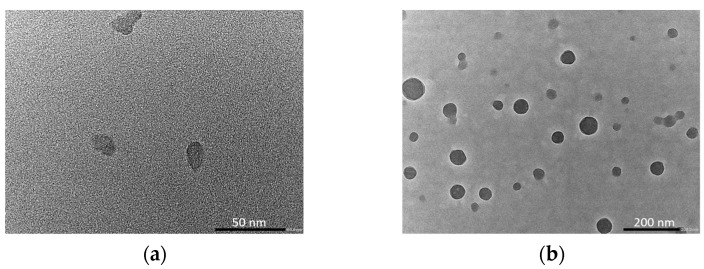
TEM image of nanoaggregates of **Ir-NH** formed in water–CH_3_CN mixtures with (**a**) 0% and (**b**) 99% water fraction.

The weak emission of Ir-NH in CH_3_CN solution may be due to the excited state relaxation caused by the distorted structure of the Ir(III) complex, which leads to an effective pathway for nonradiative decay [40,41,42]. Normally, the complex has poor solubility in water. As the water content increases, **Ir NH** may aggregate in a mixed solvent of water and CH_3_CN. The closer packing arrangements could suppress intramolecular motion and enhance emission [43,44]. To further investigate the mechanism of the AIE phenomenon, we first tested the UV-visible absorption spectra of **Ir-NH** in a mixed solution of CH_3_CN and water. It could be observed in Figure S7 that the level-off tail of the absorption spectra was gradually warped as the water content increased in the mixed system, which is the classical Mie scattering effect [45]. With an increasing proportion of undesirable solvents, molecules will gradually precipitate out of the solution and thus aggregate. Next, we further demonstrated the formation of aggregates by transmission electron microscopy (TEM) experiment. As shown in Figure 2, when the water content was 99%, the TEM photographs showed a significant increase in the number and size of the particle aggregates. Therefore, the AIE behavior mechanism of **Ir-NH** can be attributed to the aggregation of particles limiting the intramolecular motion.

### 2.3. Acid-Base Vapor Response

**Figure 3:**
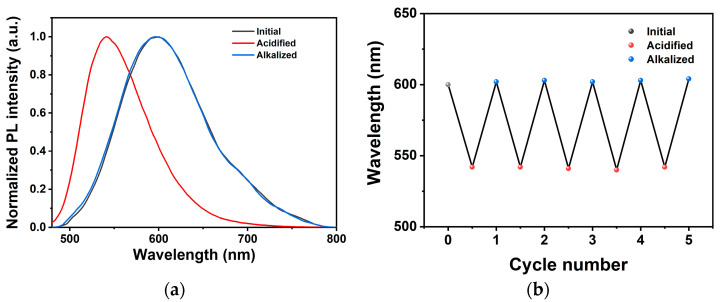
(**a**) Normalized emission spectra of the samples as indicated. Inset: emission images of initial and acidified sample under 365 nm UV illumination. (**b**) The repeated cycle of acid-base vapor stimulation process.

Because of the amino and N-heterocyclic substituents of **Ir-NH**, we conjectured that its emission properties would be altered by acid-base vapor. As shown in Figure 3a, the initial color of **Ir-NH** was orange (λ_max_ = 600 nm). When the sample was exposed to hydrochloric acid (HCl) vapor, its color rapidly changed to green (λ_max_ = 541 nm) within a few seconds, with a blue shift in the maximum emission wavelength of 59 nm. The apparent color change was easily observed by the naked eye. Interestingly, the green sample acidified by HCl vapor was then fumigated with aqua ammonia vapor, and its color quickly returned to orange, with the emission peaks recovering as before. More importantly, **Ir-NH** exhibited good cycling under acid-base vapor stimulation, due to its maximum emission wavelength remaining nearly consistent, indicating that the complex could be used as an excellent acid-base responsive material (Figure 3b).

### 2.4. Study of Acid-Base Response Mechanism

**Figure 4:**
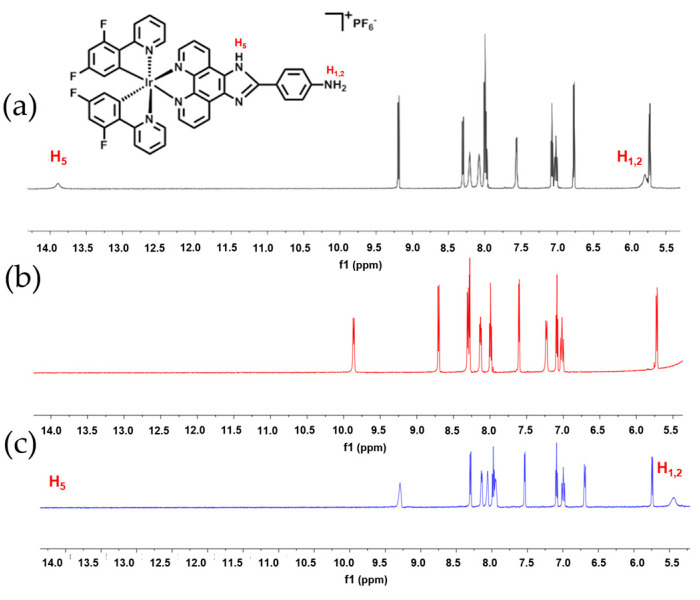
(**a**) ^1^H NMR spectrum of the initial sample of **Ir-NH**, (**b**) ^1^H NMR spectrum of **Ir-NH** with the addition of HCl (1.2 M), (**c**) ^1^H NMR spectrum with the addition of HCl followed by the addition of ammonia (2.5%).

To investigate the acid-base response mechanism of **Ir-NH**, ^1^H NMR experiments were first performed. As shown in Figure 4, compared with the initial spectrum of **Ir-NH**, the proton signals at -NH_2_ (H_1, 2_) and -NH (H_5_) positions were significantly eliminated after being acidified with HCl. It is possible that due to the action of HCl affecting the intermolecular arrangement or interactions, the chemical shifts in the proton signals at other positions were altered accordingly. When ammonia was added to the HCl-treated samples, it was observed that the disappearing proton signals at the H_1, 2_ positions reappeared (Figure 4c). The active hydrogen proton signal at the H_5_ position was not revealed because of the introduction of water. Apart from that, the NMR spectrum of **Ir-NH** was almost restored as before. Therefore, we speculated that the reaction sites for protonation and deprotonation could be the -NH_2_ and -NH substituents on the auxiliary ligand.

**Figure 5:**
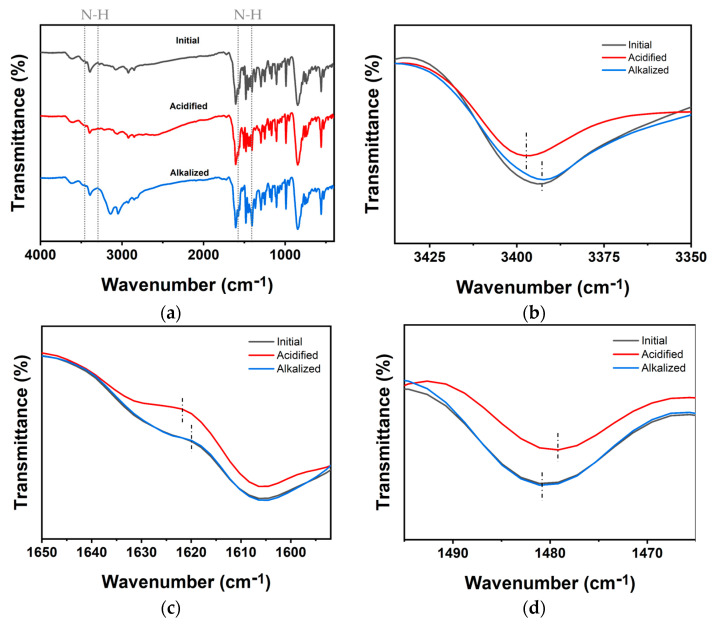
(**a**) IR spectra of **Ir-NH** after acid-base stimulation. (**b**) Stretching vibrational peaks of N-H substituents. Bending vibrational peaks of N-H (**c**) and NH_2_ (**d**) substituents.

To further determine the reaction sites where protonation occurs in the complex, we performed FT-TR spectral tests on pairs of initial, acidified, and alkalized samples, respectively. As shown in Figure 5, the peak at 3393 cm^−1^ for **Ir-NH** arose from the characteristic stretching vibration of the N-H substituent, and the peaks at 1480 cm^−1^ and 1615 cm^−1^ were generated by bending vibrations from the N-H and -NH_2_ substituents, respectively. The intensity and shifts in the -NH_2_ and -NH peaks in the spectra of the acidified samples were markedly changed. When the acidified sample was treated with ammonia vapor, the peaks of -NH_2_ and -NH of **Ir-NH** returned to agree with the initial sample. Therefore, we further confirmed the protonation and deprotonation reaction sites of **Ir-NH** by FT-TR spectra as -NH_2_ and -NH substituents in the auxiliary ligand.

**Figure 6:**
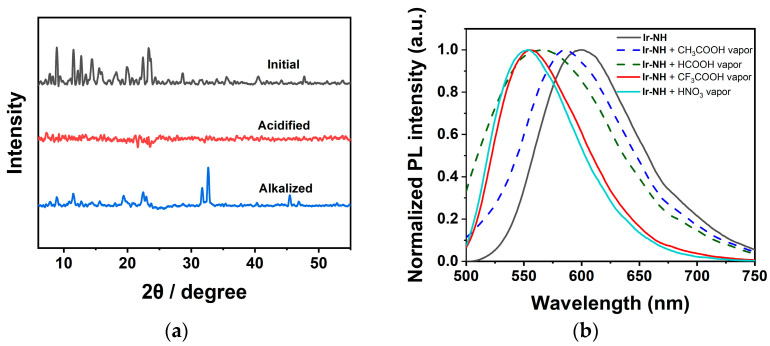
(**a**) PXRD patterns of the samples as indicated. (**b**) Normalized emission spectra of **Ir-NH** under different acid vapors at room temperature.

Finally, we carried out powder X-ray diffraction (PXRD) of the initial, acidified and alkalized samples of **Ir-NH** (Figure 6a). The diffraction peaks of the initial sample were clear and sharp, indicating that the initial state of **Ir-NH** was an ordered crystalline state. After fumigation with HCl vapor for a few seconds, the sharp diffraction peaks of the acidified sample almost wholly disappeared, which indicated that the crystalline state of **Ir-NH** was destroyed and transformed into an amorphous state. When the acidified samples were fumigated with ammonia, the sharp diffraction peaks reappeared, and the samples returned to their initial ordered crystalline stacking. Nevertheless, compared to the initial sample, a new strong peak appeared on the spectrum after alkalization. This is because the ammonium would capture protons from the already protonated N-H and -NH_2_ substituents and form ammonium chloride salts with the free chloride ions when the acidified sample was in an ammonia vapor environment, so the new sharp diffraction peaks on the spectra were attributed to the newly formed salts [46,47]. Moreover, the salt covering the surface of the molecule will prevent the crystallization of **Ir-NH** during deprotonation [48]. We have demonstrated through the above experimental data that the acid-base response mechanism of **Ir-NH** is summarized as follows. Stimulated by the acid vapor, the protonation reaction was carried out by the N-H and NH_2_ substituents in the auxiliary ligand, disrupting the initial stacking pattern of the molecule from crystalline to the amorphous state, which lead to a blue shift in the emission color from orange to green. When the acidified sample was placed in an alkali vapor environment, **Ir-NH** underwent another rapid deprotonation process, which led to the re-formation of the crystalline stack and the return of the emission color to as it was before. The main reason for the change in phosphorescence of **Ir-NH** is the protonation and deprotonation process induced by acid-base vapor stimulation, which leads to the transition from a crystalline to amorphous state.

### 2.5. Response of **Ir-NH** to Different Volatile Acid Vapors

To understand the sensing effect of **Ir-NH** on other volatile acids, we selected four common volatile acids in the laboratory, namely acetic acid (CH_3_COOH), formic acid (HCOOH), trifluoroacetic acid (CF_3_COOH), and nitric acid (HNO_3_). **Ir-NH** was fumigated in the vapor of each of the above volatile acids for a few seconds, and the changes in the emission spectra are shown in Figure 6b. The emission peaks of **Ir-NH** showed different degrees of blue shift under different acid vapors. **Ir-NH** showed the minimum change in emission wavelength in response to CH_3_COOH vapor stimulation, followed by HCOOH, probably due to their weak acidity [49,50]. The four volatile acids were ranked in order of their responsive ability as HNO_3_ ≈ CF_3_COOH > HCOOH > CH_3_COOH. The color change in **Ir-NH** in the presence of all four acid vapors was evident and could be observed by the naked eye. Thus, the complex exhibits excellent potential to be used as a sensor for detecting volatile acids.

### 2.6. Sensing Device Studies of **Ir-NH**

**Figure 7:**
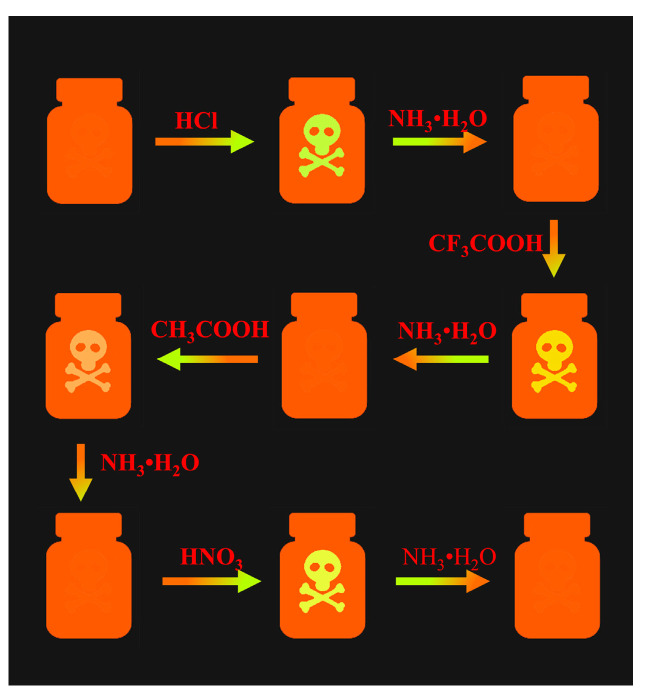
A simple and fast recyclable phosphorescent paper-based sensing device for volatile acid detection. The warning signal is based on **Ir-NH**.

Due to the excellent and reversible acid-base response properties of **Ir-NH**, we believe that the material has the potential as a phosphorescent sensor to detect volatile acids in daily production or life. For acid-base sensors, it is desirable to have the advantages of simplicity, convenience, and speed. Therefore, we constructed a simple and fast recyclable phosphorescent paper-based sensing device for monitoring volatile acids. Firstly, we selected the previously reported **complex 1 [44]**, which has an emission wavelength of 600 nm but does not have acid-base responsive properties, as the background material. The filter paper was cut into the shape of a wide-mouth bottle to make a film substrate, and **complex 1** was uniformly covered on the filter paper. **Ir-NH** was made into a warning signal (skull and crossbones) to be placed on the film, which formed a simple paper-based sensing device (Figure 7). Under UV light, the background substrate and the warning signals were orange and difficult to distinguish. When this sensor was placed in HCl environment, the volatile acid acted as a switch that quickly activated the orange warning signal to green, alerting people to the presence of volatile acid in that environment and directing them to please pay attention to their protection. It is important to note that by fumigating this device with ammonia, the green warning signal could revert to orange, and a new round of volatile acid detection could be performed. The warning signal will reappear when other volatile acids are monitored. This sensing device can be recycled many times and has good stability.

## 3. Materials and Methods

### 3.1. General Information

Reagents and solvents were used as instructed by the supplier. Unless otherwise specified, all purifications were carried out by column chromatography using 200-300 mesh silica gel supplied by the supplier. The structure of the complex was characterized by a Bruker AV600 NMR spectrometer (Bruker, Billerica, MA, USA) and a Bruker autoFlex III mass spectrometer (Bruker, Billerica, MA, USA). Ultraviolet-visible tests were conducted using a Shimadzu UV-3100 spectrophotometer (Shimadzu, Kyoto, Japan). Fluorescence emission spectra were measured by an Edinburgh FLS920 steady-state transient fluorescence spectrometer (Edinburgh Instruments Ltd., Livingston, UK). FT-TR spectral data were obtained by a 6700-FTIR Fourier Transform Infrared Spectrometer (Nicolet, USA).

### 3.2. Synthesis and Characterization of Complex

**Figure 8:**
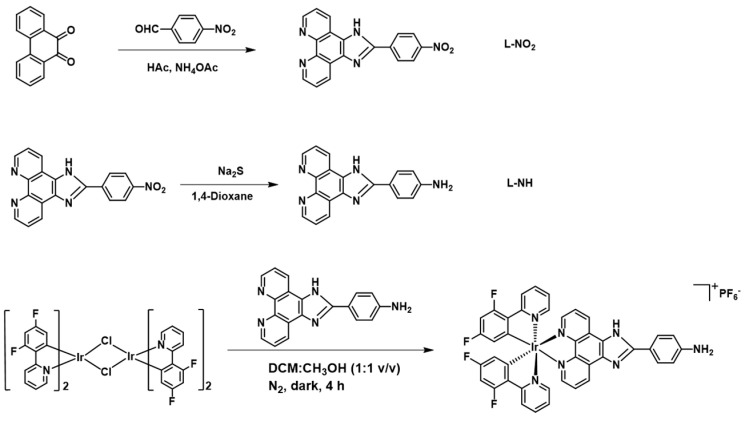
Synthesis route of **Ir-NH**.

The resultant route is detailed in Figure 8.

#### 3.2.1. Synthesis of L-NH

According to the available literature, 1,10-Phenanthroline-5,6-dione (0.4202 g, 2.00 mmol) and 4-Nitrobenzaldehyde (0.3125 g, 2.12 mmol) were first suspended in acetic acid solution (20 mL). Then, ammonium acetate (3 g) was added and stirred at 120 °C for 2 h. After the reaction, the precipitate was filtered and collected, washed with water and dried to obtain **L-NO_2_** in 90% yield. ^1^H NMR (600 MHz, DMSO) δ 14.08 (s, 1H), 9.05 (s, 2H), 8.91 (d, *J* = 8.3 Hz, 2H), 8.48 (q, *J* = 9.0 Hz, 4H), 7.85 (dt, *J* = 28.4, 7.9 Hz, 2H).

**L-NO_2_** (0.821 g, 2.34 mmol) and 1,4-dioxane (20 mL) were added to a two-necked flask and heated to 80 °C. A hot solution of sodium sulfide was added dropwise to the flask and stirred for 4 h. The yellow suspension immediately changed to a red solution. The precipitate was collected by filtration and washed with water and petroleum ether; the product **L-NH** was reddish brown in 80% yield. ^1^H NMR (600 MHz, DMSO) δ 8.96 (dd, J = 4.3, 1.9 Hz, 1H), 8.89 (dd, *J* = 8.2, 1.9 Hz, 1H), 7.99 (d, *J* = 8.4 Hz, 1H), 7.78 (dd, *J* = 8.1, 4.2 Hz, 1H), 6.72 (d, J = 8.4 Hz, 1H), 5.53 (s, 1H).

#### 3.2.2. Synthesis of Ir-NH

**L-NH** (0.062 g, 0.2 mmol) and [Ir(dfppy)_2_Cl_2_]_2_ (0.121 g, 0.1 mmol) were dispersed in a mixed dichloromethane (15 mL) and methanol (15 mL) solution, which was stirred at 78 °C under an atmosphere of N_2_ in the dark for 4 h. The mixture was cooled to room temperature and an excess of KPF_6_ was added and stirred for 30 min at room temperature. The product was then filtered and the solvent was removed under vacuum. The crude product was purified by silica gel column chromatography with dichloromethane/ethyl acetate (10:2 *v*/*v*) as the eluent, affording an orange solid. (58%). ^1^H NMR (600 MHz, DMSO) δ 13.89 (s, 1H), 9.19 (d, *J* = 8.3 Hz, 2H), 8.30 (d, *J* = 8.8 Hz, 2H), 8.21 (s, 2H), 8.07 (d, *J* = 6.4 Hz, 2H), 7.99 (q, *J* = 8.5 Hz, 4H), 7.56 (d, *J* = 6.1 Hz, 2H), 7.07 (t, *J* = 6.9 Hz, 2H), 7.02 (ddd, *J* = 12.3, 9.4, 2.5 Hz, 2H), 6.77 (d, *J* = 8.6 Hz, 2H), 5.81–5.71 (m, 4H).

## 4. Conclusions

In summary, we have rationally designed and synthesized a novel cationic Ir(III) complex. Ir-NH exhibits typical AIE properties and is an ideal phosphorescent material for acid-base stimulation reactions. Due to the introduction of N-H and NH_2_ substituents on the auxiliary ligands, **Ir-NH** undergoes protonation and deprotonation reactions stimulated by acid-base vapor, which converts the stacking state of the molecules from crystalline to amorphous state, leading to H exhibiting reversible switching of emission colors in response to acid-base stimulation. Based on this, we have prepared a simple phosphorescent paper-based sensing device, with the advantages of easy portability, fast response, and recyclability, for monitoring volatile acids in experiments or in life. This work introduced multiple acid-base response sites into iridium complexes by molecular design, resulting in fast and easy acid-base response detection. It greatly enriches the application of phosphorescent materials in solid-state sensing and provides a clear design direction for developing efficient intelligent materials.

## Data Availability

All data generated or analyzed during this study are included in this published article and its supplementary information files.

## Supplementary Materials

The following supporting information can be downloaded at: https://www.mdpi.com/article/10.3390/molecules29246041/s1. Figure S1. ^1^H NMR spectrum of **L-NO_2_** in DMSO-*d*_6_; Figure S2. ^1^H NMR spectrum of **L-NH** in DMSO-*d*6; Figure S3. ^1^H NMR spectrum of **Ir-NH** in DMSO-*d*6; Figure S4. Mass spectrum of the **Ir-NH**; Figure S5. Absorption and emission spectra of **Ir-NH** in CH_3_CN solution (1.0 × 10^−5^ M) at room temperature; Figure S6. The fluorescence lifetime of **Ir-NH**; Figure S7. Absorption spectra of **Ir-NH** in CH_3_CN-water mixtures (complex concentration = 1.0 × 10^−5^ M) with different water fractions (0–99%, *v*/*v*) at room temperature; Table S1. Photophysical data of **Ir-NH**.
