# Supplementary material for: Phosphorescent Sensor Based on Iridium(III) Complex with Aggregation-Induced Emission Activity for Facile Detection of Volatile Acids
Source: Molecules. 2024 Dec 22;29(24):6041. doi: 10.3390/molecules29246041 (PMC11677296; doi:10.3390/molecules29246041)
Supplement: Supplementary file 1 [file molecules-29-06041-s001.zip › molecules-3353681-supplementary.pdf]

Supporting information

Phosphorescent sensor based on Iridium(III) complex with AIE activity for facile detecting volatile acids

Yu Pei [1], Yan Sun [1], Dongxia Zhu [1,*]

1 Key Laboratory of Nanobiosensing and Nanobioanalysis at Universities of Jilin Province, Department of Chemistry, Northeast Normal University, 5268 Renmin Street, Changchun, Jilin Province 130024, P. R. China

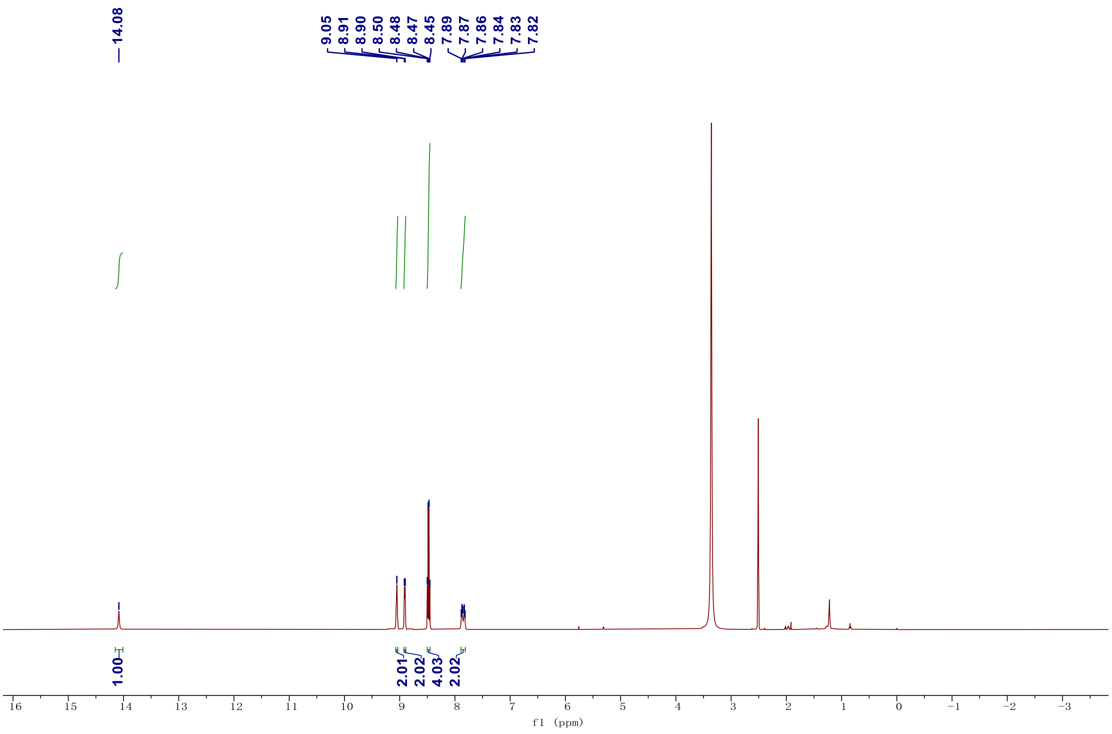

Figure S1. ^{1}H NMR spectrum of **L-NO$_2$** in DMSO-d_6.

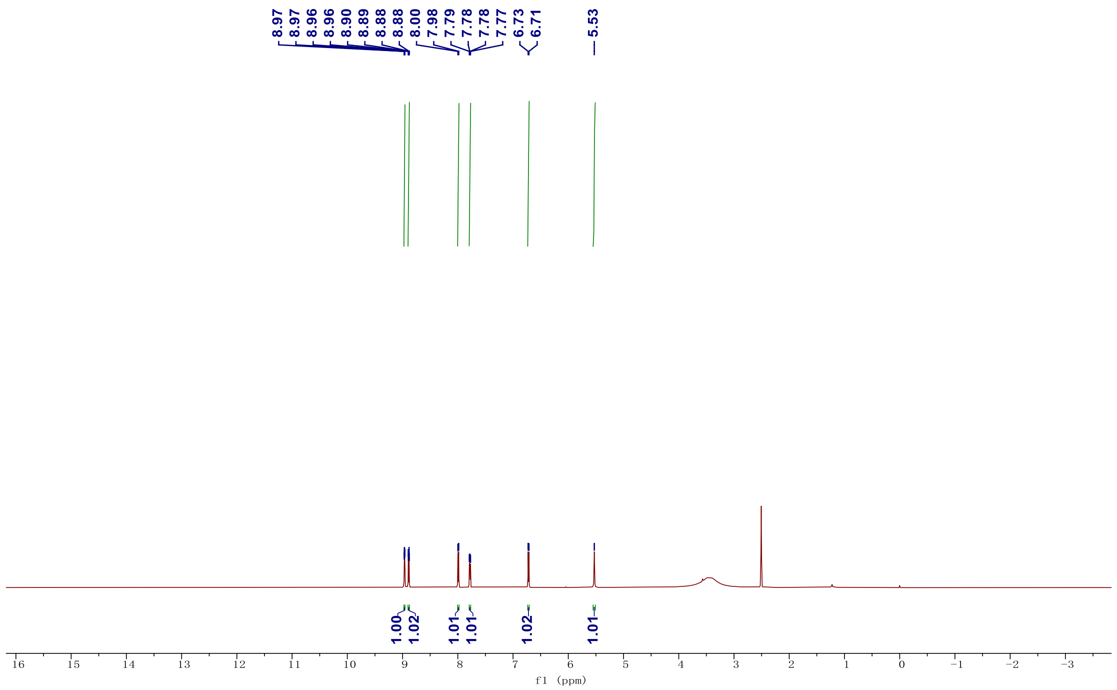

Figure S2. ^{1}H NMR spectrum of **L-NH** in DMSO-*d6*.

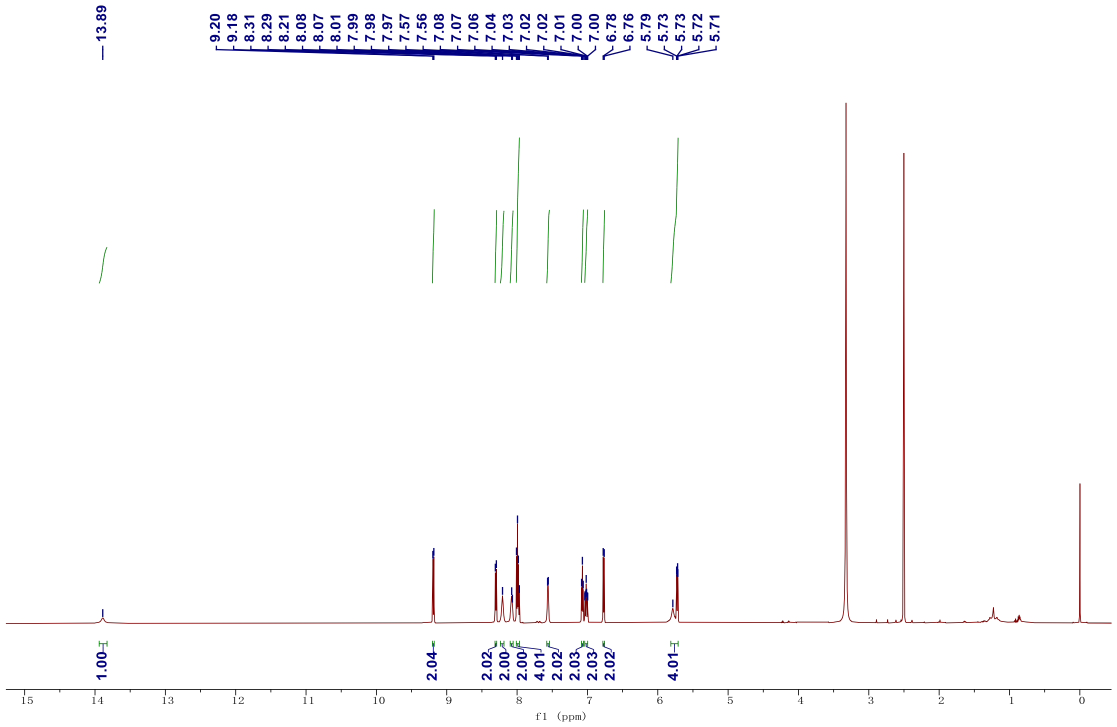

Figure S3. ^{1}H NMR spectrum of **Ir-NH** in DMSO-*d6*.

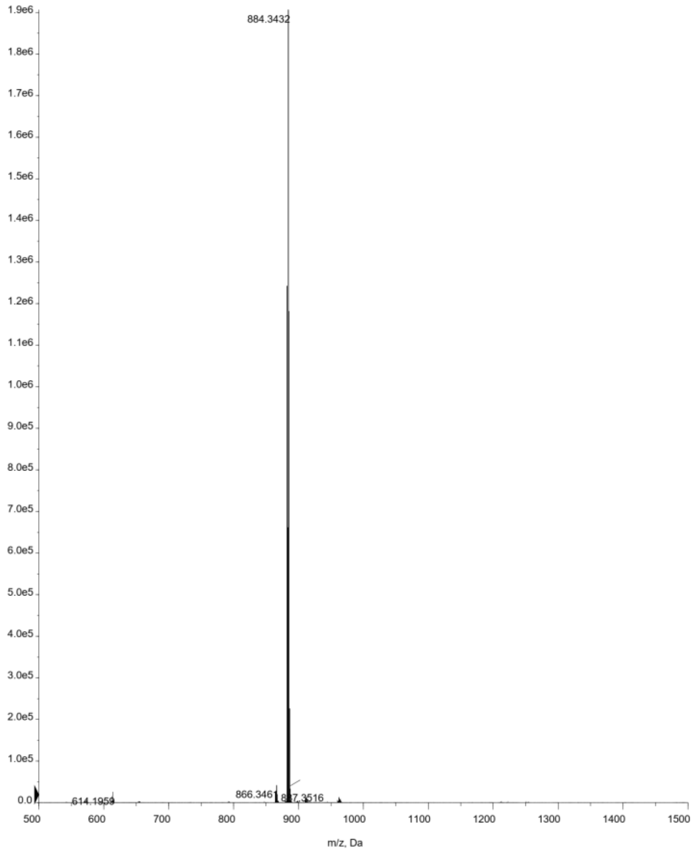

Figure S4. Mass spectrum of the **Ir-NH.**

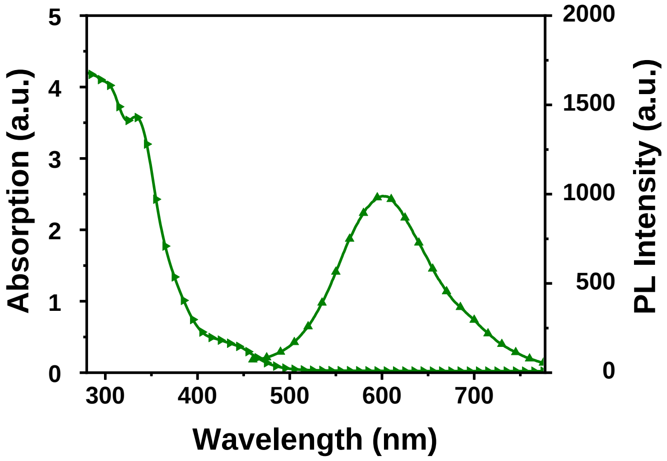

Figure S5. Absorption and emission spectra of **Ir-NH** in CH_3CN solution (1.0×10^{-5} M) at room temperature.

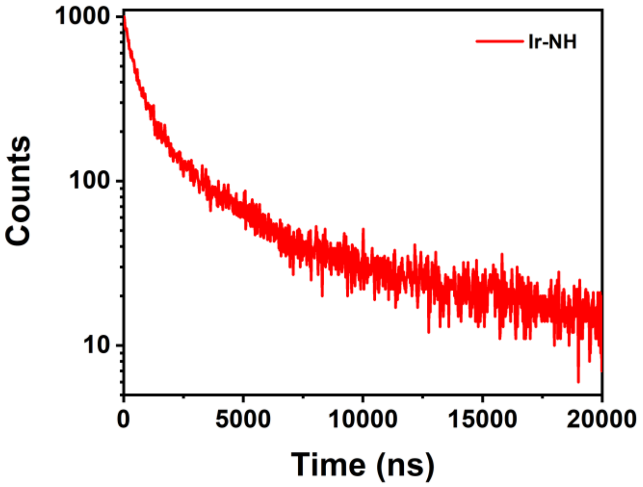

Figure S6. The fluorescence lifetime of **Ir-NH.**

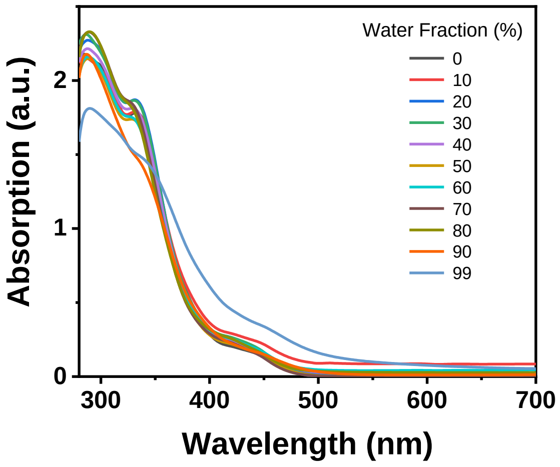

Figure S7. Absorption spectra of **Ir-NH** in CH_3CN-water mixtures (complex concentration = 1.0×10^{-5} M) with different water fractions (0–99%, v/v) at room temperature.

Table S1. Photophysical data of **Ir-NH.**

Absorption and emission at room temperature					$k_r \times 10^6$ s^{-1}	$k_{nr} \times 10^6$ s^{-1}
$\lambda_{abs}{}^a$ (nm)	$\lambda_{em}{}^a$ (nm)	$\lambda_{em}{}^b$ (nm)	$\Phi_{em}{}^b$	τ^b (μs)		
334(1.068)	602	600	0.15	2.12	0.015	8.24

[a] Measured in CH_3CN (1.0×10^{-5} M) solution. [b] Measured in solid state.
